# Evidence of Validity and Reliability of the Lasher and Faulkender Anxiety about Aging Scale in Mexican Older Adults

**DOI:** 10.3390/healthcare9121612

**Published:** 2021-11-23

**Authors:** María del Carmen Zueck-Enríquez, Ma. Concepción Soto, Susana Ivonne Aguirre, Martha Ornelas, Humberto Blanco, Jesús Enrique Peinado, Juan Cristóbal Barrón-Luján, Juan Francisco Aguirre

**Affiliations:** Faculty of Physical Culture Sciences, Autonomous University of Chihuahua, Chihuahua ZP 30000, Mexico; mzueck@uach.mx (M.d.C.Z.-E.); masoto@uach.mx (M.C.S.); mornelas@uach.mx (M.O.); hblanco@uach.mx (H.B.); jpeinad@uach.mx (J.E.P.); jcbarron@uach.mx (J.C.B.-L.)

**Keywords:** factor analysis, anxiety, aging, construct validity, structural equations, invariance

## Abstract

Anxiety about aging is an important mediating factor in attitudes and behavior toward elderly individuals as well as a mediating factor in the adjustment to one’s own aging processes. The aim of this study was to analyze the factor structure, internal consistency and factorial invariance by sex of the Lasher and Faulkender Anxiety about Aging Scale. The sample consisted of 601 Mexican older adults, 394 women and 207 men, with a mean age of 70.69 ± 8.10 years. The factor structure of the questionnaire was analyzed using confirmatory factor analysis. Analyses show that a four-factor structure is feasible and adequate. The four-factor structure (fear of the elderly, psychological concerns, physical appearance and fear of loss), according to statistical and substantive criteria, showed adequate reliability and validity indicators. However, the obtained model does not fully coincide with that proposed by the questionnaire authors, although it continues to support the multi-factor component of anxiety about aging. On the other hand, the factor structure, the factor loadings and the intercepts are considered invariant in the two populations (men and women); however, there are differences between populations on the means of the physical appearance and fear of loss factors.

## 1. Introduction

Discrimination due to age seems to be a phenomenon that is learned through exposure to social and cultural prejudices, and has implications for people’s general functioning. Currently, the media tends to stereotype and increase this feeling of marginalization and devaluation towards the stage of old age, focusing on youth and beauty and associating aging with negative attitudes [1,2,3].

Hence, such perceptions influence the way the aging process is perceived in various age groups, how older people perceive themselves, and how this makes them feel. A study by Taşdemir [4] showed that negative attitudes towards older people are associated with higher levels of anxiety about aging; in addition, some authors warn that negative beliefs and stereotypes have an impact on people’s self-perception throughout life, becoming expectations and beliefs about the aging process itself, associated with health problems that affect general well-being [5,6,7,8,9,10].

Anxiety about aging is conceptually different from trait state anxiety and anxiety about death [11] and overlaps with the concepts of psychological well-being and attitudes about aging [12]. This anxiety influences attitudes and behaviors towards the elderly and the adaptation to the aging process itself; decreased physical attractiveness and fear of looking old are associated with less optimism, fears about social identity, and greater fear of death [13,14,15].

In 1993, Lasher and Faulkender [12] developed the Anxiety about Aging Scale, pointing out that worries and anticipation about the losses that occur during the aging process constitute anxiety about aging, and that it is a mediating factor in attitudes and behaviors towards aging, the elderly and towards the adaptation to the aging process itself; such imbalance can manifest itself in four dimensions: physical, psychological and social transpersonal or spiritual. These dimensions are synthesized in three specific fears Fear of aging or the aging process itself, Fear of being an older person and Fear or anxiety towards the elderly. This questionnaire initially consisted of 84 items, but after evaluating the factor structure, it was reduced to 20 items which are distributed in four factors that explain 50.6 percent of the total variance and have a high internal consistency.

Prior research has assessed the validity of the Lasher and Faulkender [12] Anxiety about Aging Scale in older adults from various parts of the world and in diverse contexts. Although acceptable psychometric properties have been confirmed, supporting the factor structure of the original version, researchers have also encountered discrepancies in the number and meaning of the items due to participants’ age, sex and culture [16,17,18].

In Mexico there is limited research on the validity of this scale in particular for older adults, we were able to locate a single study carried out by Rivera-Ledesma et al. [19].The researchers assessed older adults with an average age of 63 years; the factor structure of the scale yielded four factors (positive attitude towards old people, fear of physical changes, old age and dissatisfaction with the self and life and old age and satisfaction with the self and life), with a general internal consistency of 0.76 and a 60.8% of explained variance; this version coincided 60 percent with the one proposed by Lasher and Faulkender [12], retaining 12 of the 20 items.

Given the importance of the anxiety about aging construct, it is essential to be able to assess it using valid and reliable instruments. For this reason, the present instrumental study [20] has aimed to provide empirical support to the factor division of the Anxiety about Aging Scale proposed by Lasher and Faulkender [12]; which is justified by the relevance of checking the factor structure of an instrument and its psychometric equivalence in different groups [21].

## 2. Materials and Methods

### 2.1. Participants

A total of 601 Mexican older adults participated in the study, 394 women and 207 men, the sample was obtained through a convenience sampling. Participant’s age ranged between 60 and 90 years, with a mean of 70.69 and a standard deviation of 8.10 years.

### 2.2. Inclusion Criteria

Participants who resided in the city of Chihuahua, aged 60 or more years, who agreed to participate in the study, and who did not have any problem that prevented them from answering the questionnaire were considered.

### 2.3. Exclusion Criteria

Participants who did not complete the questionnaire.

### 2.4. Measurement Instrument

The Lasher and Faulkender Scale of Anxiety about Aging [12]. This questionnaire is a Likert-type scale on which the participant responds on a scale of 1 to 5 to each of the proposed aspects. Higher scores indicate higher levels of anxiety about aging. The questionnaire consists of 20 items that are grouped into four dimensions of anxiety about aging (five items per dimension): (1) Fear of the Elderly: measures external contact with others (e.g., “I enjoy being with people older than me”); (2) Psychological Concerns: reflects more personal or internal problems (e.g., “I think it will be very difficult for me to feel happy when I am older”); (3) Physical Appearance: contains elements related to anxiety about changes in physical appearance (e.g., “I have lied about my age in order to look younger”); and (4) Fear of Loss: relates to loss of social support and autonomy (e.g., “I fear that when I am older all my friends will have died”). Participants indicated their agreement with each item on a five-point Likert-type scale ranging from strongly agree (1) to strongly disagree (5).

The Spanish version by Fernández-Jiménez, Álvarez-Hernández, Salguero-García, Aguilar-Parra and Trigueros [16] was used for our study; three adaptations were made prior to data collection.

For the first adaptation, the version by Lasher and Faulkender [12] is scored with five response options ranging from (1) completely disagree to (5) completely agree; on the version used in the present research, the participant chooses among 11 possible answers. We combined the original scale with our version which resulted in the following scale: Completely Disagree (0), Disagree (1, 2 and 3), Neither Agree nor Disagree (4, 5 and 6), Agree (7, 8 and 9) and Completely Agree (10).

The second adaptation consisted of changing some terms used in the items of the original version in order to use more appropriate vocabulary for the Mexican context and the age of the respondents.

**Figure 1:**
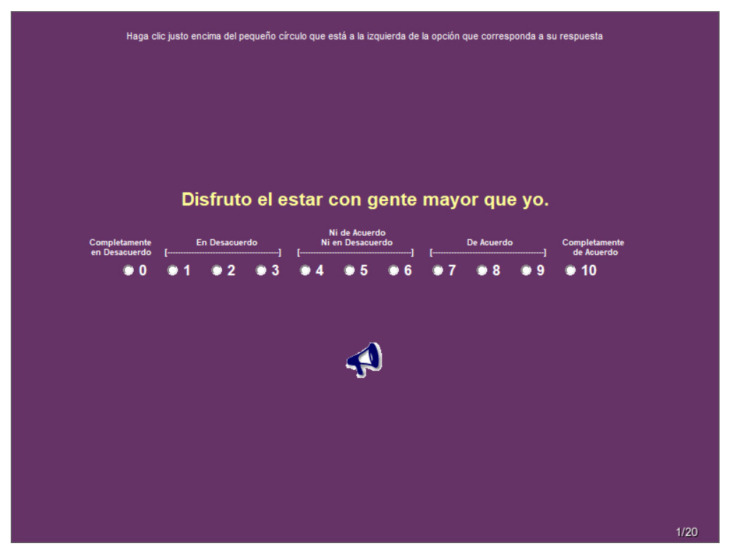
Example of response for the items of the questionnaire.

The third adaptation consisted of applying the instrument by means of a computer (Figure 1); this was done in order to allow the data to be stored with greater precision and speed, and eliminating prior coding stages.

### 2.5. Procedure

This research followed the guidelines and regulations of the Mexican General Health Law for Research on Health and followed the list of elements of free and informed consent indicated by Mondragón-Barrios [22]. In addition, the research protocol was approved by the Scientific Committee at the Department of Research and Postgraduate Studies of the Faculty of Physical Culture Sciences of the Autonomous University of Chihuahua. Older adults from the city of Chihuahua, Mexico, were invited to participate in the study. Those who agreed to participate signed the informed consent. Then, the instrument described above was completed using a personal computer, in a single, approximately 30-min session.

At the beginning of the session, a short introduction was made about the importance of research and how to access the instrument. Utmost sincerity was requested from the participants and confidentiality of the data was guaranteed. Instructions on how to respond were placed on the first screens, before the first instrument item. At the end of the session, they were thanked for their participation. Once the instrument was completed the results were collected using the results generator module of the scale editor version 2.0 [23].

### 2.6. Data Analyses

The first step in the analysis of the psychometric properties of the questionnaire consisted of calculating the mean, standard deviations, skew and kurtosis for each item. Items with extreme skew or kurtosis were later removed from the scale.

Two measurement models were compared: Model 1 (AAE-4F), a four-factor model according to the distribution of the items within the original questionnaire, and Model 2 (AAE-4Fm), which corresponds to the factor structure of the previous model, removing the items that were not sufficiently well explained by Model 1.

AMOS 21 software [24] was used to conduct the confirmatory factor analyses; the variances of the error terms were specified as free parameters, on each latent variable (factor) one of the structural coefficients was set to one so that its scale would be equal to that of one of the observed variables (items). The maximum likelihood estimation method was used following the recommendation of Thompson [25], in the sense that when confirmatory factor analysis is used, not only the fit of a theoretical model should be corroborated, but it is advisable to compare the fit indices of several alternative models in order to select the best one.

To assess the fit of the model, the Chi-square statistic, the goodness of fit index (GFI), the standardized residual mean square root (SRMR) and the root mean square error of approximation (RMSEA) were used as measures of absolute fit. The adjusted goodness of fit index (AGFI), the Tucker-Lewis Index (TLI), and the comparative fit index (CFI) were used as measures of incremental fit. The Chi-square ratio over the degrees of freedom (CMIN/DF) and the Akaike information criterion (AIC) as parsimony indices [26,27].

Next, the reliability of each dimension of the tested models was calculated using Cronbach’s alpha [28,29] and the omega coefficient [30,31].

Subsequently, in order to obtain a test that presents the best properties for the conformation of the scores of the Anxiety about Aging Scale in women and men, an analysis of the factorial invariance of the measurement models obtained for the samples of women and men was performed using the best model obtained in the total sample (AAE-4Fm model) as the baseline. Finally, the reliability of each dimension was calculated in both samples using Cronbach’s alpha and the omega coefficient [30,31].

## 3. Results

### 3.1. Descriptive Analyses

The descriptive analyses of each of the 20 items of the questionnaire show that the responses to all the items reflect mean scores that range between 1.13 and 6.92, and the standard deviation offers, in all cases, values greater than 2.20 (with a response range between 0 and 10). All skew values are within the ± 2.30 range and most kurtosis within the ± 2.50 range; so it is inferred that the variables reasonably fit a normal distribution.

### 3.2. Confirmatory Factor Analyses

The overall results of the confirmatory factor analysis (GFI 0.919; RMSEA 0.062; CFI 0.919) for the AAE-4F model indicate that the measurement model is acceptable (Table 1).

The set of the four factors of the AAE-4F model explain approximately 58% of the variance. On the other hand, 6 of the 20 items have saturations below 0.70 in their expected dimension (items 2, 4, 5, 6, 7 and 16). Low to moderate intercorrelations between the four factors are observed showing an adequate discriminant validity between them.

The overall results of the confirmatory factor analysis (GFI 0.966; RMSEA 0.051; CFI 0.976) of the second and last model tested (AAE-4Fm) corresponding to the four-dimensional structure of the previous model without items 2, 3, 4, 5, 6, 7, 16 and 19, which were not sufficiently well explained by the AAE-4F model or that according to the modification indices were not adequate, indicate that this measurement model is better than the previous model and its fit is optimal (Table 1). The four factors in this model together explain approximately 72% of the variance.

Furthermore, only two of the 12 items have saturations below 0.70 in their expected dimension (items 8 and 17). Again, low to moderate intercorrelations between the factors are observed, showing an adequate discriminant validity between them (Table 2).

### 3.3. Reliability of the Subscales (Internal Consistency) in the Total Sample

The factors obtained in the confirmatory factor analyses in both models, with the exception of the Psychological Concerns factor in the AAE-4F model, reach internal consistency values above 0.70, providing evidence for an adequate internal consistency (Table 3).

### 3.4. Confirmatory Factor Analyses for Women and Men

The results of the confirmatory factor analysis of 12 items grouped into four factors (AAE-4Fm) in the sample of women are optimal (GFI = 0.955; RMSEA = 0.058) and according to the incremental fit and parsimony indices, significantly higher to the independent model and very similar to the saturated model (Table 4). On the other hand, the confirmatory factor analysis for the sample of men indicates that the four-factor measurement model is acceptable (GFI = 0.917; RMSEA = 0.082) and according to the incremental fit and parsimony indices, significantly higher than the independent model and very similar to the saturated model (Table 4).

According to the results shown in Table 5, in both samples, most of the items saturate equal to or above 0.70 in their predicted dimension, which provides evidence of an appropriate convergent validity. Also, low to moderate intercorrelations between the factors were observed, showing an adequate discriminant validity between them.

### 3.5. Invariance of the Factor Structure between Women and Men

The fit indices (Table 6) support the equivalence of the basic measurement models among the two samples. Although the Chi-square value exceeds that required to accept the invariance hypothesis, the indices GFI = 0.941, CFI = 0.958, RMSEA = 0.047 and AIC = 345,000 contradict this conclusion, allowing us to accept the base model of the invariance (unconstrained model).

We characterize the metric invariance by imposing restrictions on the factor loadings to the baseline model. The values shown in Table 6 support this level of invariance. The general fit index (GFI 0.940) and the root mean square error of approximation (RMSEA 0.045) continue to provide convergent information in this direction. In addition, the Akaike information criterion (AIC 333.560) and the Bentler comparative index (CFI 0.959) do not vary greatly with respect to the previous model. Using the criteria for the assessment of nested models proposed by Cheung and Rensvold [32], who suggest that if the calculation of the difference of the CFI of both nested models decreases by 0.01 or less, the restricted model is considered good and therefore supporting the factorial invariance; the obtained difference between CFIs allows us to accept the metric invariance model. We can conclude so far that the factor loadings are equivalent in both samples.

Once metric invariance between the samples has been demonstrated, we proceed to assess the equivalence of the intercepts (strong factorial invariance). The indices (Table 6) show an acceptable fit of this model, both independently evaluated and analyzed with respect to its nesting within the metric invariance model. The difference between the Bentler comparative indices is 0.003; the general fit index is 0.634 and the root mean square error of approximation is 0.045. Accepting the strong invariance, the two models are found to be equivalent with respect to the factor coefficients and the intercepts.

The factors obtained in the confirmatory factor analyses, in both samples, reach internal consistency values equal to or above 0.70; evidencing an adequate internal consistency (Table 7).

### 3.6. Contrasts of Factor Means of the Samples of Women and Men

Once the factorial invariance had been verified, the differences between the means of the factors of the two groups were estimated using the sample of women as a reference, setting the value of the means for that sample to 0 and freely estimating the value of the means for the sample of men. The restrictions on the regression coefficients and intercepts, required for the contrasts between the means, were carried out automatically using the AMOS 21 software [24]. The results of the comparisons indicated that the means of the factor Fear of the Elderly is significantly higher in men (0.585, *p* < 0.01). While for the factors Psychological Concerns (0.168, *p* > 0.05), Physical Appearance (0.058, *p*> 0.05) and Fear of Loss (−0.036, *p* > 0.05), no significant differences were found.

## 4. Discussion

The goal of the present study was to obtain data on the structure and factorial invariance of the Anxiety about Aging Scale proposed by Lasher and Faulkender [12] in a sample of older Mexican women and men. Results from the analyses showed that the AAE-4Fm model with a four-factor structure: (a) Fear of the Elderly, with three items (1, 10, and 13), (b) Psychological Concerns, with two items (11 and 18), (c) Physical Appearance, with four items (9, 12, 15 and 20) and (d) Fear of Loss, with three items (8, 14 and 17), is a valid and viable structure for the Scale of Anxiety about Aging completed by Mexican older adults of both sexes. Results that, in general, are in line with those obtained by Lasher and Faulkender [12]. In addition, the factors correlate with each other in a positive and statistically significant way, which shows that as anxiety increases in one of them, it also increases in the other. In summary, this version of the Anxiety about Aging Scale has provided satisfactory data that fit the underlying theoretical model and show high consistency and validity.

However, the obtained model differs to a certain extent from that proposed by Lasher and Faulkender [12], because, in order to achieve a better fit and greater discrimination capacity, eight of the twenty items analyzed had to be removed (item 2: I fear that when I am older all my friends have died, item 3: I like to visit my relatives who are older than me, item 4: I have lied about my age in order to look younger, item 5: I think it will be very difficult for me to feel happy when I am older, item 6: When I am older my health is what worries me the most, item 7: I will have a lot to occupy my time when I am older, item 16: I think that when I am older I will still be able to do almost all things for myself and item 19: I enjoy doing things for people who are older than me). The observed discrepancies between the model proposed by Lasher and Faulkender [12] and the one proposed here can be attributed to the fact that Lasher and Faulkender included a wide variety of age groups in their sample, in contrast, the present research only includes the sample of older adults.

On the other hand, the AAE-4Fm model agrees almost entirely with that proposed by Rivera et al. [19] who included also a sample of older adults; where, as in the present investigation, eight of the twenty items of the original proposed scale by Lasher and Faulkender [12] had to be eliminated. Thus reaffirming the idea that the discrepancies observed between the model proposed by Lasher and Faulkender [12] and the one proposed here only for older adults can be attributed to the age group to which they belong.

Together with all the aforementioned, the results of the factorial invariance analysis between women and men; indicate a high congruence between the pairs of factors. This suggests the existence of strong evidence of the cross-validation of the measure and therefore of the stability of the structure, until the contrary is proven. In addition to the fact that the comparisons between the groups reflected significant differences in one of the factors (Fear of the Elderly), which seems to indicate that older adult men in comparison with their female counterparts, tend to present higher levels of anxiety about aging in relation to the anxiety generated by external contact with others. Which, in general, agrees with findings reported by Aguirre, et al. [33] who state that women show more anxiety in relation to decision-making or the loss of the meaning of life as an older adult, while men do so in relation to living with older adults.

## 5. Conclusions

The factor structure obtained in the present investigation, due to the number of items and their theoretical coherence with the original version by Lasher and Faulkender, can be considered a short and adapted version of the Anxiety about Aging Scale for use in older adults.

Finally, it should be mentioned that the scope of these results is limited, and it is necessary that future research replicates the obtained structure, which will allow for more robust evidence regarding the factor structure of the questionnaire. It is considered that more studies are necessary in order to corroborate or refute the data obtained in the research carried out so far.

It is also essential to check whether the questionnaire is useful, for example, in predicting psychological well-being. It will also be important that the scale can be interpreted on the basis of norm-referenced scores (e.g., percentiles).

## Figures and Tables

**Table 1:** Absolute, incremental, and parsimony indices for the generated models.

Model	Absolute Indices					Incremental Indices				Parsimony Indices	
	χ^2^	GFI	RMSEA	SRMR		AGFI	TLI	CFI		CMIN/DF	AIC
AAE-4F	537.745 *	0.919	0.062	0.066		0.896	0.906	0.919		3.279	629.745
AAE-4Fm	122.261 *	0.966	0.051	0.032		0.945	0.966	0.976		2.547	182.261

Note: * *p* < 0.05; GFI = goodness of fit index; RMSEA = root mean square error of approximation; SRMR = standardized residual mean square root; AGFI = corrected goodness-of-fit index; TLI = Tucker-Lewis index; CFI = comparative fit index; CMIN/DF = chi-square ratio over degrees of freedom; AIC = Akaike Information Criterion.

**Table 2:** Standardized solutions from the confirmatory factor analyses for AAE-4F y AAE-4Fm models.

Item	AAE-4F					AAE-4Fm			
	F1	F2	F3	F4		F1	F2	F3	F4
Factor Loadings									
1 I enjoy being with people who are older than me	0.79					0.79			
3 I like to visit relatives who are older than me	0.66					-			
10 I enjoy talking with people who are older than me	0.86					0.86			
13 I feel very comfortable around someone who is older than me	0.86					0.86			
19 I enjoy doing things for people who are older than me	0.72					-			
5 I think that it will be difficult for me to feel happy when I am older		0.36					-		
7 I will have a lot to occupy my time when I am older		0.53					-		
11 When I am older I think I will feel good about life		0.80					0.82		
16 I think that when I am older I will still be able to do almost everything by myself		0.43					-		
18 When I am older, I trust that I will feel good about myself		0.76					0.77		
4 I have lied about my age in order to appear younger			0.39					-	
9 It bothers me to imagine myself being older			0.72					0.71	
12 It worries me that when I am older I will see more wrinkles when I look in the mirror			0.74					0.73	
15 Seeing myself older has worried me			0.76					0.77	
20 When I look in the mirror, it bothers me to see how my appearance has changed with age			0.74					0.72	
2 I fear that when I am older all my friends will have died				0.50					-
6 When I am older, my health is what worries me the most				0.45					-
8 I get nervous when I think that someone else will make decisions for me when I am older				0.67					0.65
14 I worry that when I am older people will ignore me				0.73					0.73
17 I worry that when I am older life will lose its meaning				0.68					0.68
Factor Correlations									
F1	-					-			
F2	0.56	-				0.52	-		
F3	0.40	0.51	-			0.39	0.50	-	
F4	0.15	0.46	0.76	-		0.18	0.42	0.78	-

Note: F1 = Fear of the Elderly, F2 = Psychological Concerns, F3 = Physical Appearance, F4 = Fear of Loss.

**Table 3:** Omega and alpha coefficients for the factors obtained from the confirmatory factor analyses for the AAE-4F y AAE-4Fm models.

Factor	AAE-4F			AAE-4Fm	
	Ω	α		Ω	α
Fear of the Elderly	0.89	0.88		0.88	0.87
Psychological Concerns	0.72	0.69		0.77	0.78
Physical Appearance	0.81	0.81		0.82	0.83
Fear of Loss	0.75	0.74		0.73	0.73

**Table 4:** Absolute, incremental, and parsimony indices for the generated models. Confirmatory factor analyses for women and men.

Model	Absolute Indices				Incremental Indices				Parsimony Indices	
	χ^2^	GFI	RMSEA		AGFI	TLI	CFI		CMIN/DF	AIC
Factor solution for women										
AAE-4Fm	111.230 *	0.955	0.058		0.926	0.956	0.968		2.317	171.230
Saturated	0.000	1.000					1.000			156.000
Independent	2057.981 *	0.400	0.277		0.291	0.000	0.000		31.182	2081.981
Factor solution for men										
AAE-4Fm	113.769 *	0.917	0.082		0.865	0.918	0.940		2.370	173.769
Saturated	0.000	1.000					1.000			156.000
Independent	1165.746	0.402	0.284		0.294	0.000	0.000		17.663	1189.746

Note: * *p* < 0.05; GFI = goodness of fit index; RMSEA = root mean square error of approximation; AGFI = corrected goodness-of-fit index; TLI = Tucker-Lewis index; CFI = comparative fit index; CMIN/DF = chi-square ratio over degrees of freedom; AIC = Akaike information criterion.

**Table 5:** Standardized solutions from the confirmatory factor analysis for the AAE-4Fm Model. Samples of female and male participants.

Item	Women					Men			
	F1	F2	F3	F4		F1	F2	F3	F4
Factor Loadings									
1 I enjoy being with people who are older than me	0.77					0.80			
10 I enjoy talking with people who are older than me	0.90					0.81			
13 I feel very comfortable around someone older than me	0.83					0.89			
11 When I am older I think I will feel good about life		0.83					0.84		
18 When I am older, I trust that I will feel good about myself		0.76					0.77		
9 It bothers me to imagine myself being older			0.70					0.75	
12 I worry that when I am older I will see more wrinkles when I look in the mirror			0.72					0.77	
15 Seeing myself older has worried me			0.78					0.76	
20 When I look in the mirror, it bothers me to see how my appearance has changed with age			0.72					0.74	
8 I get nervous when I think that someone else will make decisions for me when I am older				0.68					0.61
14 I worry that when I am older people will ignore me				0.77					0.67
17 I worry that when I am older, life will loose its meaning				0.67					0.70
Factor Correlations									
F1	-					-			
F2	0.51	-				0.56	-		
F3	0.45	0.52	-			0.32	0.46	-	
F4	0.25	0.42	0.74	-		0.07	0.42	0.85	-

Note: F1 = Fear of the Elderly, F2 = Psychological Concerns, F3 = Physical Appearance, F4 = Fear of Loss.

**Table 6:** Goodness of fit indices for each of the models for which factorial invariance was assessed.

Model	Fit Indices						
	χ^2^	gl	GFI	NFI	CFI	RMSEA	AIC
Unrestricted model	225.000 *	96	0.941	0.930	0.958	0.047	345.000
Metric Invariance	229.560 *	104	0.940	0.929	0.959	0.045	333.560
Strong factorial invariance	249.262 *	114	0.934	0.923	0.956	0.045	333.262

Note: * *p* < 0.05; GFI = goodness of fit index; NFI = normed fit index; CFI = comparative fit index; RMSEA = root mean square error of approximation; AIC = Akaike Information Criterion

**Table 7:** Omega and alpha coefficients for the factors obtained in both samples.

Factor	Women			Men	
	Ω	α		Ω	α
Fear of the Elderly	0.87	0.87		0.87	0.87
Psychological Concerns	0.77	0.77		0.79	0.79
Physical Appearance	0.82	0.82		0.84	0.84
Fear of Loss	0.75	0.74		0.70	0.70

## Data Availability

Data available upon request from correspondence author.
